# Adapting an Outpatient Psychiatric Clinic to Telehealth During the COVID-19 Pandemic: A Practice Perspective

**DOI:** 10.2196/22523

**Published:** 2020-10-01

**Authors:** Farzan Sasangohar, Major R Bradshaw, Marianne Millen Carlson, James N Flack, James C Fowler, Diana Freeland, John Head, Kate Marder, William Orme, Benjamin Weinstein, Jacob M Kolman, Bita Kash, Alok Madan

**Affiliations:** 1 Center for Outcomes Research Houston Methodist Hospital Houston, TX United States; 2 Behavioral Health Clinic Houston Methodist Hospital Houston, TX United States

**Keywords:** telemedicine, psychiatry, preventive psychiatry, SARS virus, pandemic, prevention, COVID-19, telehealth, perspective

## Abstract

As the demand for telepsychiatry increases during the COVID-19 pandemic, the strengths and challenges of telepsychiatry implementation must be articulated to improve clinical practices in the long term. Currently, observations within US contexts are lacking; therefore, we report on the rapid implementation of telepsychiatry and workflow experiences in a psychiatric practice based within a large health care system in southeast Texas with a national catchment area. We discuss the logistics of the implementation, including modes of communication, scheduling, coordination, and capacity; the psychological effects of web-based services, including both the loss of the physical therapeutic environment and the unique interpersonal dynamics experienced in the virtual environment; and postadoption patterns of engagement with our services and with other clinical functions affected by the rapid adaptation to telemedicine. Our art therapy group programming serves as an applied case study, demonstrating the value of a well-managed web-based program (eg, patients were receptive and well-engaged, and they appreciated the continuity of accessible service) as well as the challenges (eg, the need for backup plans and technological fallbacks, managing interruptions and telecommunication learning curves, and working around the difference in resources for art and music therapy between a well-stocked clinical setting versus clients’ home spaces). We conclude from our experience that the overall strengths of telepsychiatry include receptive and well-engaged responses from patients as well as the expansion of boundaries, which provides a directly contextualized view into patients’ home lives. Challenges and corresponding recommendations include the need for more careful safety planning for high-risk patients; maintaining professional boundaries in the newly informal virtual setting; designing the physical space to both frame the patient encounter and maintain work-life balance for the therapist; allowing for delays and interruptions (including an initial acclimation session); and preserving interprofessional care team collaboration when the physical locations that normally facilitate such encounters are not accessible. We believe that careful observations of the strengths and challenges of telepsychiatry during this pandemic will better inform practices that are considering telepsychiatry adoption both within pandemic contexts and more broadly thereafter.

## Introduction

The spread of COVID-19 and wide-scale self-quarantine and shelter-in-place orders have led many nonemergency practices to adopt telehealth solutions to continue serving their patients [1], and psychiatry is no exception. While some cases will still warrant inpatient psychiatric admissions, including coordinated care for psychiatric inpatients who test positive for COVID-19 [2], telepsychiatry is a viable option for handling outpatient scenarios. Best practices are already in place [3], and there is a supportive evidence base for telepsychiatry [4-6], particularly for web-based therapy for depression [7] and posttraumatic stress disorder [8]. However, the forced adoption of and mass transition to telehealth during the COVID-19 pandemic has resulted in significant challenges for the implementation of telehealth for psychiatric services [9,10]. While guidelines exist for postpandemic telepsychiatry [11,12], given the unique characteristics of this COVID-19 pandemic, lessons learned from these transitions as well as implementation-specific guidelines would be highly applicable to other practices. To date, clinical implementations and lessons learned (and with largely positive results) have been reported largely from settings outside the United States, such as China [13,14], Europe [15-23], Turkey [24], and Australia [10,25-28], with only some US coverage (notably [9], [29], and [30]). In this paper, we document our experience with telehealth adoption at a psychiatry practice embedded in a large health system in the United States and summarize several important successful improvisations and challenges to support broader and more diverse adoption efforts.

Established in January 2018, the psychiatric outpatient clinic at Houston Methodist Hospital offers programs of care to address gaps in the current mental health treatment continuum. The therapeutic outpatient assessment services span approximately 10 business days (2 to 4 hours per day) and are tailored to patients with complex and persistent psychiatric conditions, including depression, anxiety-related disorders, trauma, psychosis, interpersonal dysfunction, chronic pain, sleep disorders, emotional regulation problems, and suicidality. The team-based, multidisciplinary assessment is designed to optimize insight, produce maximal diagnostic clarity, and create a roadmap for future treatment.

The clinic offers access to mental health care through a >5-week Functional Rehabilitation Program that includes morning group therapy sessions and individual sessions in the afternoon. The program proceeds on an outpatient basis, enabling patients to complete treatment while remaining engaged with their lives. The approach to treating patients incorporates an understanding of broader contexts, such as medical comorbidities and social determinants of health. The clinic also offers services to meet the varied and dynamic needs of our patients, including an intensive outpatient program, a modified functional rehabilitation program, individual psychotherapy, couples counseling, psychotropic medication management, and art and music therapies.

In some ways, our clinic had a “head start” on the COVID-19 pandemic compared to most other clinics, as it was already transitioning to digital platforms before the pandemic [31]. The system-wide support for innovative technologies in our health system had resulted in early adoption of and significant investments in telehealth, including secure and integrated technology to facilitate virtual visits embedded within our electronic health record (EHR), a virtual intensive care unit [32], and adoption of telemedicine postoperative follow-up [33]. We had already vested and integrated a platform called CareSense (MedTrak, Inc) for previsit and postvisit care coordination and patient-reported outcomes (see [31] and Fowler et al, forthcoming). Hence, frequently cited sources of provider hesitation to adopt telemedicine, including workflow integration and infrastructure-related logistics [4,34-37], did not apply to our practice or to our overall culture. However, the COVID-19 pandemic still necessitated rapid adoption of changes to go live before full testing.

## Implementation and Experiences

On March 18, 2020, our outpatient clinic transitioned to a 100% telehealth platform, and it is continuing with this approach as of this writing. Our complete transition and continued use of telehealth technologies contrasts with many other outpatient practices, where clinics have followed suit with local businesses and have opened to provide at least some in-person treatment. We chose a more conservative approach to the ongoing use of telehealth technologies for many reasons. Hospital-wide policies require face masks and six feet of social distancing for all outpatient visits for both patients and providers. These safety requirements reduce the risk of COVID-19 transmission but come at a significant cost to the daily practice of outpatient psychiatric care. Our assessments and interventions rely heavily on nonverbal and verbal communication. Masks cover well over half of patients’ faces, limiting providers’ assessment of patient affect. Additionally, masks muffle voices. This facet of mask-wearing has the potential to negatively affect the content of verbal output and subsequently affect comprehension for patients and providers alike. Verbal communication from at least six feet away only worsens the potential negative consequences of muffled voices.

### Diversifying Modes of Communication: One Size Does Not Fit All

In our practice, we found it essential to leverage multiple platforms and modalities (eg, Cisco Webex, Microsoft Teams, email, telephone calls, the EHR, patient portal communications) to facilitate the initial transition. While such an inclusive strategy may increase the logistical complexity of care for the providers, our clients found the backup options and redundant systems to be essential. For instance, one client became anxious and frustrated by initial difficulties downloading Webex; therefore, we pivoted to using FaceTime (Apple Inc), which did not require an additional tool and was familiar to the client. Another client, who was feeling lonely and disconnected but struggled to find time for sessions because of childcare demands, was able to feel connected by occasionally texting her therapist. The texts included YouTube links to humorous videos on shared pandemic struggles. In addition to serving a wide range of client preferences, this flexibility was key to help us maintain the availability of service even when one modality lagged or became unresponsive. In addition, having different levels of fidelity on hand (eg, from high-tech Webex to low-tech telephone calls) created a scaffold for backup options and platforms that can be used in tandem. Of course, a list of both COVID-19–provisional approved applications and technology that is more generally compliant with the Health Insurance Portability and Accountability Act (HIPAA) should be consulted to ensure digital security and privacy [38].

Conversely, diversifying the modes of communication also carries efficiency costs (both within the care team and in team-client interactions). These include logging in to platforms and waiting for others to do the same; pausing for deliberate transition between speakers; and reporting the same information in different forms and in multiple places (eg, rapid switches between sessions on Microsoft Teams and formal documentation of the same in the EHR).

### Special Logistical Concerns

Handoff procedures were particularly transformed in the new process. Using the gaps between sessions for quick handoffs results in moving from meeting to meeting without a break and then ending the day with more in-depth documentation. We experienced an increased need for communication between team members; casual hallway or “water-cooler” handoffs frequently occur in physical clinic settings, but these could not occur when the care team shifted to telecommuting. Some team members adapted to the loss of this natural and implicit means of coordination by more explicit efforts, while others “went silent.” We learned that staff should consciously expect and prepare for these easily underestimated differences in communication dynamics.

Management of schedules and appointment times was also more stressful and demanding, as tardiness or absence from appointments became ambiguous. An office no-show can occur for many reasons but at least is always counted as an absence. Web-based absence can result from an otherwise ready client experiencing technical difficulties, a broken or misdirected link, or an adjacent appointment; other no-show reasons include forgetfulness or distractions due to other turmoil (quarantine-related or otherwise). In addition, team members’ and clients’ free time has been transformed, limited or expanded, and distributed differently due to lockdown. Factors such as childcare, other caregiver needs, or the presence or absence of other adults in the home have become factors that affect schedules.

Finally, we found that team members and clients had variable capacity to formulate their communication behaviors and subsequently adapt them to the virtual leap. Technological comfort, literacy, and fluency also played roles. When finally online, users experienced differences in the conversational flow in the web-based environment and had to adjust their interviewing styles, such as avoiding talking over the patient. Video exists in a liminal space between in-person interaction and nonvisual interaction. When face-to-face, interlocutors experience familiar nonverbal cues to signal when a speaker is finished or when a listener is still attentive or becoming unsettled, which informs the turn order of speaking as needed; in radio communications, there is a clear expectation that these cues will be absent (hence, conventions are in place such as saying “over” to replace the cues). Web-based video lacks the fidelity to retain the cues but “teases” users with at least some visual context; therefore, we may not properly reset our expectations (nods are missed in choppy video feeds, pitch cues are missed in the audio transmission, eye contact is disrupted by the offset camera eye, etc.). Delays in communication as speakers are confused by the turn order affect the length and efficiency of the session, and more time is required to build rapport and trust with new patients.

### Psychological Effects of Web-Based Audiovisual Chat

Our clinic emphasizes differentiation through “time and team” to produce a therapeutic effect. With telehealth, we need to be more mindful of the “time” aspect, which as noted above has been transformed. Web-based chat fatigue (also referred to as “Zoom fatigue” during the COVID-19 pandemic) of patients and team members has sometimes motivated us to limit the duration and frequency of sessions to spread out the mental effort required. Patients still receive the time that our clinic promises; however, they may need to be more flexible with the duration of our programs. As a team, we do not have as much time to talk about patients together because we are confined to team meetings and have strict cutoff times and schedules before the next Webex meeting begins. We also miss the in-person consultations between staff, which have social and not purely logistical benefits. For both our clients and health care workers, a well-established period of reflective *time* can be as therapeutic and rejuvenating as any sacred or holy *space* [39]; therefore, the fragmentation of that time into asynchronous processes such as emails is felt. We have used other technology formats and call or text providers as needed to communicate. The needed information is communicated; however, these consultations are quick and detailed information is lost.

Time and space coalesce to form a therapeutic environment for mental health. Space is now virtual, with sessions taking place in the patient’s home or home office in a manner contiguous with the rest of their day. Thus, they can lose the effect of relocating to a separate and unique space, such as the psychiatrist’s office. In this new space, team members and clients have needed to work harder to generate and sustain interpersonal connection. Social signals and efforts must be amplified to overcome the loss of intimacy and belonging derived from physical proximity. This is difficult when new relationships begin in the virtual environment, in contrast to existing clients, with whom rapport was already established before the transition to virtual sessions. Conversely, video chat garners some closeness because of the virtual invitation into normally private spaces (eg, the therapist’s and client’s home office or kitchen table). Viewers obtain unintended but inevitable glimpses into each other’s home lives (children, pets, spouses roaming in the visual or audio background) [40], and even dress tends to be less formal.

### Services and Service Lines Under Telehealth

We carefully scrutinized which services can transition without an unacceptable loss of integrity and which cannot. Individual therapy is an easy and established service for telehealth [5,7]; group therapy has been received well by providers and clients alike, perhaps because the program is small. Art and music therapy programs had precedents; however, the success of transition was uncertain, and exploratory efforts were required to establish workability, as in our case study below. Neuropsychological services had to be limited to simple screening. These choices were determined by individual workplaces but should not arise in a vacuum.

Some programs may thrive more during quarantine than they otherwise would via telemedicine or other modes. Namely, we suspect that people are currently happy to connect in group programs because they are not seeing anyone else due to quarantine. Although there are still some technological challenges, patients are glad to have a social space. Once clients re-engage with their normal social supports and are not quarantined, participation in group sessions may decrease.

Specific functions of our psychiatric practice have also been transformed. Some psychological testing can now be completed through vendor-supplied web-based portals. Other tests require a physical presence, such as medical tests, genetic testing, and magnetic resonance imaging (MRI), and therefore present a challenge. More time is needed to order these tests and to receive results. We no longer have the luxury of ordering and coordinating care within our system. We must rely on external facilities, and relevant information is not always readily available in our EHR. Releases of information and other paper forms must be completed by hand, scanned by patients, and then emailed to us. Fillable forms (eg, smart PDFs) are more efficient and easier for patients who may lack access to technology or suitable home office supplies.

## Case Study: Art/Music Therapy Group

Art and music therapies are integral components of our uniquely structured Functional Rehabilitation Program; all patients participate in these forms of therapy in both individual and group settings. While more common forms of psychotherapy are also features of the core clinical programming (eg, acceptance and commitment therapy, dialectical behavioral therapy skills training, process-oriented group therapy), we chose to highlight art and music therapy because of the additional logistical challenges these therapies required in transitioning from in-person sessions to a telehealth platform—namely, the need for therapy-specific equipment, such as painting supplies and musical instruments.

The first tele–art therapy group was held by telephone rather than video due to technical difficulties. While it was difficult to conduct an art therapy group session without being able to see anything, the planned session included a writing prompt, and patients were able to share what they had written along with a description of what they had started to create (demonstrating the *importance of backup plans and backup technologies* noted above). The timing was a challenge as well because the session took a long time to get started due to technical issues; however, the patients were open to continuing to work on the art pieces on their own, and they brought their pieces to the next session (*anticipating in-session time spent on technology; managing interruptions*). By the second session, the video was functional, and the patients were able to show their images. The idea of using the group session as the time to start a piece and then continuing to work on the piece or create additional images during the week was well received, with mixed participation (*patients as receptive and well-engaged*). Availability of art materials is another challenge; some people have plentiful supplies at home, while others have more limited supplies. Ordering items for delivery now faces delays in shipping for many items and low stock due to the increase in the numbers of people who are quarantined or sheltering in place who are ordering art supplies (*setting the in-home therapeutic space*).

Our providers observed that the patients seemed grateful to have the groups as a way to create structure in their days and to connect with others. Pre-existing rapport noticeably helped in this case: while similar online offerings are available, there is a sense of community for existing patients that they would not find by selecting a different offering, such as an online support group or art class. At the same time, our staff served as an anchor that enabled the patients to reach out to other support groups and engage in more opportunities during this time than they might normally have done because they knew they could check in with us about the other things they were trying.

Despite positive outcomes, the applied and physical nature of art and music therapy poses unique challenges, largely revolving around the resources necessary for traditional session plans. The therapist and patient do not have equal and shared access to instruments. If a patient wants to use music education in their therapy, such as learning guitar skills, they do not have access to the music therapist’s instruments to achieve this. Time lags in internet video conferencing also make joint music-making difficult. This removes real-time improvisation and some re-creative approaches from the therapist’s repertoire until technological adjustments or advancements are made; for example, music-making interventions such as group drumming are removed from the music therapist’s toolbox.

In this type of therapy, there is also an increased burden on the patient to learn technological skills to participate in telemedicine. Even if the therapist has the resources to provide high-quality video and audio, that quality can be lost if the patients’ technological resources are not equivalent. Higher-quality technology resources are needed to ensure quality experiences more than in conversation-based interventions (which, as we’ve seen, can default to mechanisms as simple as the telephone). USB microphones and high-speed home internet are not ubiquitous even among fairly privileged patients, raising additional concerns of potentially exacerbating disparities for lower-income patients and their care access to telemedicine programs of this nature. For patients who can obtain the necessary resources, technical literacy may still present a barrier; therefore, the therapist serves a dual role as the patient's information technology support in troubleshooting not only the teleconferencing method in general but also the audiovisual equipment required.

Although not ideal for re-creative or improvisational models, the internet and its associated technologies are rich in opportunities for receptive and compositional methods of music therapy. Most technology platforms (which are free but also require a certain amount of internet bandwidth) enable screen sharing and high-quality video for receptive music therapy interventions. This includes screen sharing for collaboration on lyrics or looking up chord charts on the internet. These web-based techniques take time to learn; therefore, the therapist should budget extra time accordingly for self-orientation and patient orientation. It is important for the therapist to test their new technology skills before entering sessions. The therapist should practice their new skills with a fellow therapist or family member. Free and easy-to-learn software programs are available for music production and composition, such as GarageBand and Audacity. Granted, “easy-to-learn” is relative to the more complex software available as well as the patient’s or therapist’s baseline technological skills, although learning and improving one’s self-efficacy can have benefits and rewards as well.

## Summary of Strengths, Challenges, and Recommendations

Our clinical team identified several strengths and key challenges when moving forward with our rapidly implemented telemedicine program; these may be instructive both to other psychiatric practices that are thinking of adopting telemedicine and to practices seeking to improve currently implemented programs.

### Strengths

#### Patients Are Receptive and Well-Engaged

All our prepandemic established patients continued to engage in some degree of programming when we transitioned from an in-person platform to telehealth. We were mindful of individual differences in the extent to which patients were willing to transition to telehealth programming and in their preferred platforms. Patients and providers engaged in a shared decision-making process, and we tailored our programming and mode of service delivery based on patient preferences. Postpandemic patients generally chose a reduced intensity of treatment from the beginning of their treatment with us. Overall, most patients seemed relieved that in the midst of the pandemic, therapeutic structures and support were still accessible. Rapport is enhanced by a sense that both providers and patients are going through a crisis together; this humanizes the providers to the patients while also giving providers an opportunity to model intentional coping, balanced responses to the crisis (on most occasions), and active anxiety regulation in real time. Our experience is corroborated by other US sites that implemented telemedicine rapidly [9] as well as by generally positive reviews showing patient satisfaction with telepsychiatry [5,6].

#### Virtual Groups Have Been Well-Attended, and Engagement in Some Respects Has Increased

The relative isolation that group members are feeling has spurred them out of their comfort zones to engage more actively and depend more on their peers. For some group members, especially those with more avoidance and social anxiety, the current circumstances are leading them by necessity to experience their peers as a new and positive source of support.

#### Telepsychiatry Expands the Boundaries of Psychological Intervention Into the Real World

By necessity, telehealth visits take providers out of the one-on-one environment of traditional brick-and-mortar practice and into their patients’ everyday lives, living rooms, and backyards. In one session, we were actually able to see one patient relate with her son in real time instead of just talking with her about their interaction and interpreting it as filtered through her perception. This direct and contextualized interaction increases the ecological validity and relevance of therapy and opens opportunities for more flexible service delivery models in future practice that are more effective and helpful for patients and less restrained by antiquated “red tape.”

### Challenges and Recommendations

#### High-Risk Patients

High-risk patients require more active safety planning, case management, and tolerance of anxiety and uncertainty related to unknown aspects due to lack of in-person visits. For our established chronically suicidal patients, we made emergency contact details readily available prior to scheduled sessions, expressed the ability and willingness to engage emergency medical services if necessary, and provided direct access to our inpatient psychiatric unit should the need arise. Fortunately, we did not need to mobilize any of these contingencies for prepandemic established patients. However, for patients who were admitted to our practice after we transitioned to an exclusively virtual platform, risk management created significant logistical challenges. We did facilitate inpatient psychiatric admissions with significant assistance from family members, who needed to bring their loved ones to the hospital emergency department, were not allowed to accompany them past that point, and were required to communicate with the inpatient care team almost exclusively by telephone. Additionally, we supplemented our outpatient programming with home health aides out of medical necessity. Management of risk in a virtual setting requires considerably more time, effort, and creative problem-solving than in-person clinical care [20]. To date, we have managed risk without adverse events. In this area, further research on the process, patient and provider experience, and outcomes is warranted.

#### Establish a Provider Culture of Telemedicine Adoption

One observed strength was not a strength of our telemedicine program per se but of provider willingness to adopt telemedicine as a solution; 100% of providers transitioned to telemedicine platforms with varying degrees of fluency and need for ongoing logistical support. Studies have reported provider hesitation to adopt telemedicine based on concerns about workload [34], usability [35,36], EHR and systems integration [36], other logistics related to time and staff [37], and incentives, reimbursements, and regulations [35,36], especially in the case of telepsychiatry, which deserves special consideration when COVID-19–based federal policy changes and leniencies expire [4,41]. Hence, the success of telemedicine adoption is greatly facilitated by investing in the cultural, logistical, and infrastructural factors to enable success (see also Kalin et al [29], who likewise credit their successful COVID-19–related telepsychiatry adoption to organizational culture and prior technological readiness). However, “from-scratch” implementation is also possible, as in the case reported by Yellowlees et al [9], who impressively planned a complete program to adapt in-person sessions to telepsychiatry in one day and had fully implemented it for all patients within three working days. Other attempts were more encumbered by technical and training difficulties; however, it was found, as in our study, that use of multiple platforms had a mitigating effect [30].

#### Set the Therapeutic Frame With Intentionality to Manage Risk

The transition to a virtual frame risks blurring normal therapeutic boundaries by decreasing the formality of the encounter. For example, there is a need to demarcate a telephone-based therapy appointment from a telephone call with a friend or a fellow citizen in the midst of a pandemic. In an office, this frame is set through physical elements, including the office, furniture, and waiting room. In virtual space, this frame must be instituted verbally, though not rigidly, through maintaining intentionality about how the conversation relates to the patient’s overall goals. Mindfulness and management of the professional practitioner-patient relationship remains as important as ever. It is also worth noting that most web platforms allow for anonymous telephone calls or web-calling using a web-based profile, which allows the therapist to avoid disclosing their own personal account or telephone number when checking in with the patient.

#### Setting the Physical and Visual Backdrop Requires Forethought and Design

Setting the frame requires more than verbal and behavioral monitoring. Although it can be humanizing for the patient to see the therapist in their natural habitat, it may be necessary to hide unattractive objects in the therapist’s home. A foldable standing blind can be useful for hiding items such as laundry baskets, beds, and the kitchen counter. The blind also provides a consistent, professional, and aesthetically pleasing therapeutic environment. Lighting is also a consideration; for webcasting, it is better to be lit from the front than from the back. To avoid additional glare from the ceiling fan, the laptop computer or camera should be positioned so that the ceiling fan is not in view.

#### Maintain Work-Life Balance by Separation of Space and Time

The blinds and other physical rearrangements needed for the therapeutic space can also benefit the therapist as a transitory demarcation of work versus home spaces. Home is a refuge from work, and working from home blurs the roles of the home and work environments. Using a blind to “set up” the office changes the environment from home to work and vice versa at the end of the day. This transition helps to “give back” the space to the therapist and acts as an affordance to return to normal use of the space. If the therapist’s floor plan allows for a permanent home office space, this can also demarcate a work zone from a home zone; however, the lack of physical actions and motions for setup and breakdown in this case should be replaced by disciplined scheduling to signal work time and free time in some other way. It is worth noting that health care workers, including psychiatrists and mental health workers, can also benefit from receiving telepsychiatry services [24].

#### Introduce the Client to the Virtual Space and Expect to Take Up Initial Session Time

When beginning initial sessions, it is important to allow time for orientation to the technology [9]. In the same way a new patient may need help to find their way into the building and park their car, they may need our help to orient to the technology. It is important for the therapist to take a “walking with” stance with the patient and express that the therapist and the patient are “in this together.” Allowing time for technology and normalizing it as part of the orientation to therapy can help assuage a new patient’s anxiety about their first session. The therapist should aside a generous 10 or 15 minutes simply to help the patient with the technology. This also has the effect of building trust between the patient and the therapist. Provider-guided orientation may thus be uniquely warranted in telepsychiatry in contrast to other forms of telemedicine, in which the provider and patient may both prefer to save time by using pre-session tutorials or medical assistant–led guidance to use the technology instead.

#### Interruptions May Disrupt Session Plans and Will Require Management

Potential interruptions naturally arise as the patient's family or pets enter the picture, particularly if home childcare is an issue for either the patient or the therapist. These interruptions are inevitable and must be managed. In group sessions, this may cause patients to miss key discussions. Interruption recovery techniques include video replay (if sessions are recorded) or history logs (if time-stamped real-time notes are taken) [42]. Privacy issues can be mitigated through sound localization. Headphones are fairly essential, as they prevent family members from overhearing confidential session details or comments by therapy group members. Headphones also signal to others that the patient is absorbed in a task. If a meditative or focused exercise is planned, such as guided imagery or progressive muscle relaxation, it is important to let patients know this ahead of time so that they can take steps to help their family understand that they cannot be disturbed (and establish a backup plan in case of internet connectivity disruptions). Therapists and patients alike can also look into any affordable or employer-provided childcare options they feel safe with during the pandemic to mitigate childcare-related interruptions while remaining consistent with their household’s social distancing plan.

#### Shelter-in-Place Restrictions Challenge Active Interprofessional Collaboration

Clinical isolation highlights the value of in-person informal consultation and colocation of a multidisciplinary team, both to preserve benefits to the patient derived from such team support [20,24] and for the morale of the team itself. Having short conversations in the hall, dropping by a colleague’s office for a minute, and eating lunch with colleagues keep providers apprised of shared clinical work. Without these channels, more active time and participation are needed. We have implemented additional scheduled consultation meetings to account for the loss of informal consultation.

### Conclusion

The circumstances of the COVID-19 pandemic have created unprecedented challenges that are taxing the physical and mental health of the populace; however, the pandemic has also created unprecedented opportunities to learn, teach, innovate, and evaluate telepsychiatry strategies as necessity spurs their adoption.

## Abbreviations

EHR: electronic health record
HIPAA: Health Insurance Portability and Accountability Act
MRI: magnetic resonance imaging
